# Krüppel-Like Factor 4 Transcriptionally Regulates TGF-β1 and Contributes to Cardiac Myofibroblast Differentiation

**DOI:** 10.1371/journal.pone.0063424

**Published:** 2013-04-30

**Authors:** Yi Zhang, Ying Wang, Yan Liu, Nanping Wang, Yongfen Qi, Jie Du

**Affiliations:** Beijing Anzhen Hospital, Capital Medical University, The Key Laboratory of Remodeling-related Cardiovascular Diseases, Ministry of Education, Beijing Institute of Heart Lung and Blood Vessel Diseases, Beijing, China; University of Kentucky, United States of America

## Abstract

Angiotensin II (Ang II) plays a major role in the pathogenesis of cardiac fibrosis in hypertension. It is known that Ang II induces TGF-β1 expression. How transcription mediates Ang II-induced TGF-β1 expression, as well as its contribution to cardiac fibrosis, is unknown. We studied the role of Krüppel-like family transcription factors in Ang II-induced myofibroblast formation. We found that among the Krüppel-like family members, Krüppel-like factor 4 (Klf4) was the highest expressed form in isolated cardiac fibroblasts after Ang II treatment. Klf4 increased expression of α-SMA and collagen, as well as increased myofibroblast formation. ChIP assays showed that Klf4 specifically bound to the TGF-β1 promoter. Deletion and mutagenesis analysis showed that the sites at −184∼−180 bp and −45∼−41 bp in the TGF-β1 promoter were responsible for Klf4 transactivation of the TGF-β1 promoter. Our studies demonstrate that Klf4 plays a pivotal role in Ang II-induced cardiac myofibroblast differentiation and collagen synthesis through transcriptional upregulation of TGF-β1.

## Introduction

A growing body of evidence supports that the renin-angiotensin system is a critical mediator of cardiac remodeling and dysfunction in various cardiovascular diseases [1], [2]. Under angiotensin II (Ang II) activated conditions, morphological fibroblast-to-myofibroblast differentiation enhances. Myofibroblast is a characteristic of tissue repair, remodeling and fibrosis, commonly identified by expression of α-smooth muscle actin (α-SMA) [3]. In hypertension the emergence and persistence of myofibroblast is thought to express a set of fibrotic genes that contribute to a progressive profibrotic state [4]. It is now accepted that Ang II induces cardiac fibrosis by stimulating transforming growth factor-β1 (TGF-β1), becoming an essential molecule in fibroblast-to-myofibroblast differentiation [5]. TGF-β1, after binding to its receptors, activates downstream mediators that lead to classic Smads signaling and exerts its fibrotic effects by promoting myofibroblast differentiation as well as excessive synthesis and deposition of the extracellular matrix (ECM) [6], [7].

Ang II activates its receptor, and downstream signaling ultimately leads to increased expression of transcription factors, including the early growth response gene-1 (Egr-1), NF-κB, c-myc and the AP-1 complex [8]–[12]. Activated transcription factors may play a role in fibrosis by regulating the expression of fibrotic genes. AP-1 was first reported to increase TGF-β1 mRNA levels by a strengthened AP-1 DNA binding capacity [13]. Recently, erythroblastosis virus E26 oncogene homolog-1 (ETS-1) has been inferred to be a direct mediator of renal profibrotic effects in an Ang II infusion model through the upregulation of TGF-β1 [14]. However, how transcriptional programming regulates fibrosis in response to elevated Ang II remains unclear.

Krüppel-like factors (Klfs) are a subfamily of the zinc finger class of DNA- binding transcription factors. The three zinc fingers are usually found at the C terminus of the protein and bind to either a CACCC element or GC-box. These factors regulate gene expression and is responsible for regulating cell proliferation, differentiation, development and programmed death [15]. Klfs are important regulators in the pathogenesis of various diseases, including cardiovascular remodeling [16]–[19]. Given the essential effects of Klfs on multiple events that are associated with fibrosis, the role of Klfs in regulating TGF-β1 signaling and myofibroblast differentiation remains to be determined.

In the present study, we examined the expression of Klf family members in response to Ang II infusion in hearts. Klf4 expression was the highest Klf family member expressed. Elevated Klf4 promotes differentiation of cardiac fibroblasts to myofibroblasts. Mechanistically, Klf4 binds to the TGF-β1 promoter region and activates TGF-β1 transcription, which leads to ECM synthesis in myofibroblasts. Taken together, the results of our study demonstrate that Klf4 plays a pivotal role in regulating TGF-β1 signaling and Ang II-induced cardiac myofibroblast differentiation.

## Materials and Methods

### Isolation of cardiac fibroblasts and cardiomyocytes

Neonatal cardiac fibroblasts and cardiomyocytes were isolated from C57BL/6 WT mice (1–2 days old). Isolation was performed as we previously described [20]. Briefly, ventricular tissues were minced into small pieces in a mixture of 0.2% collagenase type II (Gibco by Invitrogen, Carlsbad, CA) for 10 mins at 37°C with agitation until the tissues were completely digested. The dispersed cells were cultured on tissue culture plates for 90 mins, and unattached cells were removed. Non-myocyte cells that attached to the dishes were cultured in Dulbecco's modified Eagle's medium (DMEM) supplemented with 10% FBS. This procedure yielded cell cultures that were almost exclusively fibroblasts by the first passage. The unattached viable cells were incubated in culture dishes for an additional 90 mins to remove unattached cells, and the attached population was rich in cardiomyocytes and was cultured on gelatin-coated dishes at 37°C in DMEM supplemented with 10% fetal bovine serum (FBS) and cytosine 1-β-d-arabinofuranoside (Sigma-Aldrich; St. Louis, MO; 10 μmol/L) to inhibit fibroblast proliferation.

### VSMCs Cultures

Mouse aortic VSMCs were isolated from thoracic aorta of C57BL/6 WT male mice. Briefly, excised thoracic aorta was washed in ice-cold 1× phosphate buffer saline (1× PBS) solution, and then ventricular media was minced into small pieces by use of scissors in a mixture of 0.2% collagenase type I for 20 mins at 37°C with agitation. The digestion buffer was replaced 3 times, at which point the tissues were completely digested. The dispersed cells were incubated in culture dishes for 2 hrs, and then removed unattached cells. VSMCs were grown in DMEM supplemented with 10% FBS and then placed in free-serum medium for 24 hrs before treated with Ang II (1 μmol/L) or PBS control.

### Adenoviral Vectors and Infection

The method for Klf4 adenovirus construction was performed as previously described [21]. The expression of the inserted Klf4 cDNA is driven by a 7×tet/minimal cytomegalovirus promoter that was further under the control of an artificial tetracycline-responsive transactivator (tTA). The adenoviruses were purified by cesium chloride methods. For adenovirus-mediated gene transfer, confluent cardiac fibroblasts were exposed to adenoviral vectors at a multiplicity-of-infection rate of ∼80–100 for 2 hrs (Ad-tTA was co-infected to induce tetracycline controllable expression). After the viruses were washed off, infected cells were further incubated for the indicated time in the presence or absence of tetracycline for 24 hrs before PBS or Ang II treatment.

For in vitro deletion of Klf4, adult cardiac fibroblasts were isolated from floxed Klf4 mice and were infected with adenovirus encoding Cre recombinase (Ad-Cre) at a multiplicity of infection (MOI) of 100. Adult WT cardiac fibroblasts infected with Ad-Cre were used as a control. To rescue Klf4 expression, Ad-Cre, Ad-tTA and Ad-Klf4 were co-infected and treated with or without tetracycline for 24 hrs before treatment.

### Klf4 Small Interfering RNA Transfection

siRNA for Klf4 and scrambled siRNA were purchased from Invitrogen (Carlsbad, CA). Using Lipofectamine RNAiMax (Invitrogen), the siRNA was transfected into mouse cardiac fibroblasts plated in 35-mm culture dishes at a final concentration of 20 nM in 2 ml of culture medium. After 24 hrs of siRNA transfection, the cells were treated with PBS or Ang II. siKlf4 was performed with the following region-specific siRNAs: siKlf4 1:5′-CCUCCUGGACCUAGACUUUdTdTAAAGUCUAGGUC CAGGAGGdTdT-3′; siKlf42:CCAAGAGUUCUCAUCUCAAdTdTUUGAGAU GAGAACUCUUGGdTdT-3′; scrambled siRNA (negative control): 5′-UUC UCCGAAC GUGUCA CGUTTACGUGACACGUUCGGAGAATT-3′.

### Plasmids, Transfection, and Reporter Assay

A genomic fragment consisting of the 5′ flanking promoter region and part of exon 1 (−250 to +37 bp) of TGF-β1 was obtained by PCR using mouse genomic DNA. The promoter fragment was subcloned into a pGL3 basic luciferase reporter vector (Promega; Madison, MI) to generate pGL3-TGF-β1-Luc. Mutations of −45∼−41 bp, −184∼−180 bp or both sites within the potential KLF-binding motif were introduced by PCR to generate pGL3-TGF-β1 mutKLF. Cardiac fibroblasts were seeded in 12-well plates (2×10^5^/well) and transfected with the vectors. The cells were incubated for an additional 24 hrs in 5% CO_2_ at 37°C. After washing, the cells were harvested in 200 μl/well of lysis buffer (Dual Luciferase Kit; Promega), and luciferase activity was measured following the manufacturer's protocol and normalized to renilla luciferase activity [22].

### Chromatin Immunoprecipitation Assay

ChIP assays were performed as previously described [23]. Briefly, 2×10^7^ NIH 3T3 cells were stimulated with Ang II (1 μmol/L) for 4 hrs prior to crosslinking for 10 mins with 1% formaldehyde, and chromatin samples were immunoprecipitated using an anti-Klf4 antibody (Santa Cruz Biotechnology) or control goat IgG antibody (Sigma-Aldrich). DNAs isolated from ChIP assays were analyzed by semiquantitative polymerase chain reaction; values were presented as relative to DNA input. PCR was performed with the following region-specific primers: for the mouse TGF-β1 promoter −184∼−180 bp Klf-binding site, 5′-TCACCGGCTTTAGTAG TGCTC-3′ and 5′-GGGGGCACTGTCTTCATCT-3′; for the −45∼−41 bp Klf-binding site, 5′-ACGCTAAGATGAAGACAGT-3′ and 5′-GCTGTCTGGAGGATCCGC-3′; for the mouse GAPDH exon 7 (the site that does not contain Klf-binding motifs; negative control) 5′-GTGGACCTC ATGGCCTACAT-3′ and 5′-GGCCTCTCTTGCTCAGTGTC -3′.

### Neutralizing TGF-β1 in cardiac fibroblasts

To analyze the effects of neutralizing TGF-β1 in Klf4 overexpressed or non-overexpressed cardiac fibroblasts, either anti-TGF-β1 neutralizing antibody (R&D Systems; Minneapolis, MN; 1.0 μg/mL) or control mouse IgG1 (Sigma-Aldrich; 1.0 μg/mL) was added.

### Klf4-floxed mice

Klf4-floxed mice were bred at the Beijing Anzhen Hospital Affiliated to Capital Medical University. The generation of Klf4-floxed mice is described in detail elsewhere [24].

### Ethics Statement

The investigations conformed to the US National Institutes of Health Guide for the Care and Use of Laboratory Animals (publication no. 85–23, 1996) and were approved by the Animal Care and Use Committee of Capital Medical University.

### RNA Preparation and Real-Time RT-PCR

Total RNA was extracted by the Trizol reagent method (Invitrogen), and first strand cDNA was synthesized with SuperScript II (Invitrogen). The transcript levels were detected by real-time PCR analysis. RT PCR amplification was performed on iQ5 Real-Time PCR Detection System (Bio-Rad,Hercules, CA) with SYBR Green JumpStart^TM^ Taq ReadyMixTM (Takara, Japan). The expression level of each gene was normalized to that of β-tubulin mRNA, which served as an endogenous internal control. The sequences of the PCR primers were shown in Table S1.

### Western Blot

Fresh cells were harvested with lysis buffer (20 mM Tris, pH 7.5),1 mM EDTA,150 mM NaCl, 1 mM EGTA, 1 mM β-glycerophosphate, 1% Triton X-100, 2.5 mM sodium pyrophosphate,1 mM Na3VO4, 4 mg/ml aprotinin, 4 mg/ml leupeptin, 4 mg/ml pepstatin, and 1 mM PMSF). Protein samples were separated by 10% SDS-PAGE. The blots were incubated with the primary antibodies anti-GAPDH (1∶3000 diluted in TBS-T, Kangwei, China), anti-Klf4 (1∶400, Santa Cruz Biotechnology), anti-α-SMA (1∶1000 dilution, Sigma-Aldrich), anti-p-Smad3 (1∶1000 dilution, Cell Signaling Technology, Beverly, MA) or anti-TGF-β1 (1∶500 dilution, Santa Cruz Biotechnology), then with IR Dye-conjugated secondary antibodies (1∶5000,Rockland Immunochemicals, Gilbertsville, PA) for 1 hr. Images were quantified by use of the Odyssey infrared imaging system (LI-COR Biosciences Lincoln, NE). The protein contents were normalized to the level of GAPDH. All experiments were repeated at least 3 times.

### ELISA Assay

To analyze secretion of TGF-β1, cardiac fibroblasts were co-infected with Ad-tTA and Ad-Klf4 or transfected with siKlf4 and placed in fresh serum-DMEM medium (10% FBS) with Ang II stimuli or PBS control after 24 hrs. After an additional 24 or 48 hrs, the concentration of active TGF-β1 in the culture medium was measured using an enzyme-linked immunoassay kit (R&D Systems; Minneapolis, MN) following the manufacturer's instructions.

### Immunohistochemistry

Cells were incubated with the primary antibodies for α-SMA (1∶200) at 4°C overnight and with FITC-conjugated secondary antibodies (Molecular Probes, Carlsbad, CA) at room temperature for 1 hr. Sections were viewed with a confocal laser scanning microscope (TCS 4D, Leica; Heidelberg, Germany) and a Nikon Labophot 2 microscope equipped with a Sony CCD-IRIS/RGB color video camera.

### Statistical Analysis

Data are expressed as the mean +/− SEM. Differences between groups were analyzed by Student's t-test or ANOVA, as well as Newman-Keuls Multiple Comparison Test using Graphpad Software (GraphPad Prism version 5.0 for windows, Graphpad Software). P<0.05 was considered to be statistically significant.

## Results

### Klf4 expression is increased in hearts in response to Ang II infusion

**Figure 1:**
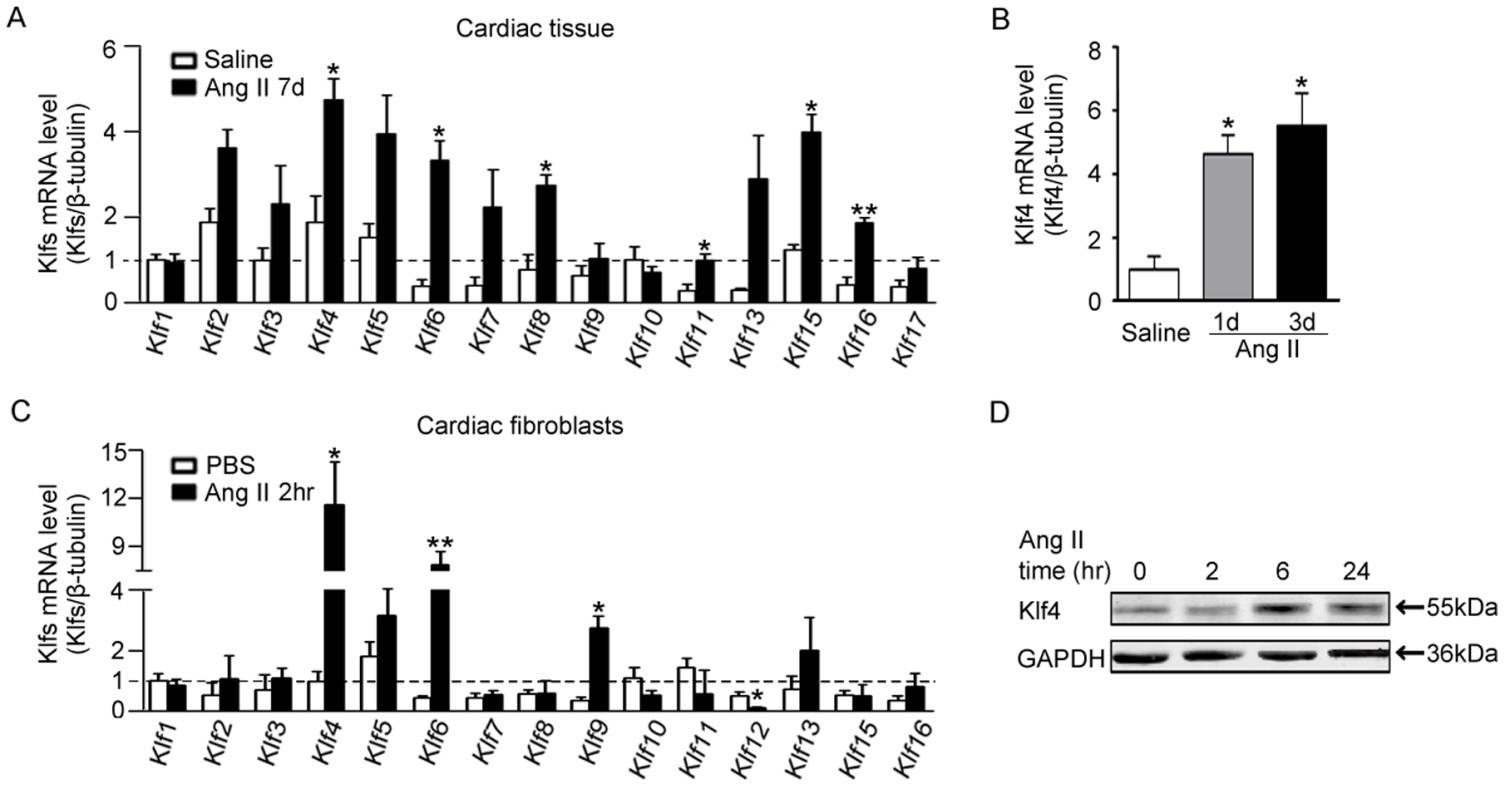
Klf4 expression is regulated in hearts in response to Ang II infusion. A, Klf mRNA levels in C57BL/6J wild-type (WT) hearts after 7 days of saline or Ang II infusion (1500 ng.kg^−1^.min^−1^). mRNA levels were assessed by quantitative real-time PCR (qPCR) and normalized to β-tubulin. Klf12 and Klf14 were not detectable in hearts. Klf17 was not detectable in saline-infused hearts (n = 3). B, qPCR of Klf4 mRNA levels in WT hearts after 3 and 5 days of Ang II infusion (n = 3). Data are the mean ± SEM. *P<0.05 vs. saline-infused control. C, Klf mRNA levels in cardiac fibroblasts after 2 hrs of stimulation with PBS control or Ang II (1 μmol/L). Klf14 and Klf17 were not detectable in cardiac fibroblasts (n = 3). D, Western blot of Klf4 protein levels after Ang II treatment. GAPDH was the loading control. Data are the mean ± SEM. *P<0.05, **P<0.01 vs. PBS control.

To determine whether Klf family members are involved in Ang II-induced cardiac fibrosis, quantitative RT-PCR (qPCR) analyses were performed to examine the expression of Klf1-17 in hearts at day 7 after Ang II infusion. Compared with saline-treated mice, Ang II infusion differentially increased the expression of KLF family members (Figure 1A). The expression of Klf4 was particularly higher. Klf4 mRNAs were significantly higher at day 1 or 3 after Ang II infusion (Figure 1B). To determine the main cellular type for Klf4 expression further, neonatal cardiomyocytes, fibroblasts or VSMCs were isolated and treated with Ang II (1 μmol/L). Klf4 expression in primary cardiomyocytes was not changed (Figure S1A) and decreased in VSMCs (Figure S1B). However, Klf4 mRNA expression was significantly increased by Ang II treatment in fibroblasts (Figure 1C). Similarly, the protein level of Klf4 was elevated after Ang II treatment at 6 hour and ebbed after 24 hrs (Figure 1D). Thus, the dynamic expression of Klf4 in cardiac fibroblasts may be involved in Ang II-induced cardiac remodeling.

### Elevated Klf4 involves Ang II-induced fibroblast activation and collagen synthesis

The expression of α-smooth muscle actin (α-SMA), a marker of myofibroblasts, was examined in cardiac fibroblasts stimulated by Ang II. Cardiac fibroblasts exhibited a low level of α-SMA. Treatment of cardiac fibroblasts with Ang II significantly stimulated α-SMA (Figure S1C). It has been reported that Ang II causes late Smad3 activation through a TGF-β1-dependent mechanism [25]. Ang II treatment also stimulates phosphorylation of Smad signaling in cardiac fibroblasts, as phosphorylated Smad3 was increased at 12 hrs and last to 48 hrs (Figure S1C), indicating activation of TGF-β1/Smad signaling in cardiac fibroblasts in response to Ang II.

**Figure 2:**
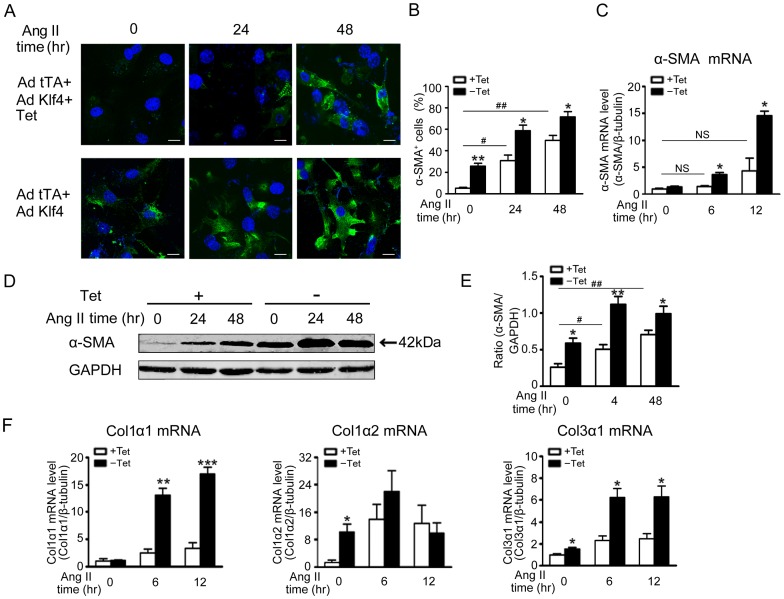
Elevated Klf4 promotes Ang II-induced fibroblast differentiation and collagen synthesis. Cardiac fibroblasts were exposed to co-infection of Ad-tTA and Ad-Klf4 for 2 hrs. After the viruses were washed off, infected cells were further incubated for the indicated time in the presence or absence of Tet (tetracycline, 0.1 μg/ml) for 24 hrs, then the cells were treated with Ang II (1 μmol/L). A, immunofluorescence analysis of myofibroblast differentiation by staining with a α-smooth muscle actin (α-SMA) antibody after Ang II treatment for 0, 24 or 48 hrs. Scale bars: 10 μm. B, Quantitative analyses of α-SMA positive cells are presented (n = 4). C, α-SMA mRNA levels were assessed by quantitative real-time PCR (qPCR) after Ang II treatment for 0, 6 or 12 hrs and normalized to β-tubulin (n = 4). D, α-SMA protein levels were assessed by Western blot after Ang II treatment for 0, 24 or 48 hrs. GAPDH was a loading control. E, Level of quantification ofα-SMA as a ratio of GAPDH in densitometric units was presented. n = 4. F, Col1α1, Col1α2, Col3α1 mRNA levels were assessed by quantitative real-time PCR (qPCR) after Ang II treatment for 0, 6 or 12 hrs and normalized to β-tubulin. n = 4. Data are the mean ± SEM. *P<0.05, **P<0.01, ***P<0.01 vs. control. ^#^P<0.05, ^##^P<0.01 vs. control and Ang II treatment for 0 hr.

To explore the role of elevated Klf4 in Ang II-induced fibrosis, an adenovirus containing the CMV-driven tet-controlled transactivator (Ad-tTA) and an adenovirus containing tetracycline-regulated Klf4 (Ad-Klf4) were used to transfect cardiac fibroblasts. There was a dose-dependent increase in Klf4 expression in adenovirus-infected cardiac fibroblasts when treated with tetracycline (Figure S2). When Klf4 was overexpressed, there was a sustained increase in the number of α-SMA positive cells in response to 24 hrs or 48 hrs of Ang II treatment (Figure 2A&B). The level of α-SMA mRNA was also increased at 6 and 12 hrs of Ang II treatment (Figure 2C), whereas the level of α-SMA protein exhibited a similar increase in Klf4-overexpressed cells at both 24 hrs and 48 hrs (Figure 2D&E). Expression of Klf4 in cardiac fibroblasts increased the expression of type I and III collagen (Col1, Col3). As shown in Figure 2F, mRNA levels for Col1α1, Col1α2 and Col3α1 were increased in response to Ang II.

**Figure 3:**
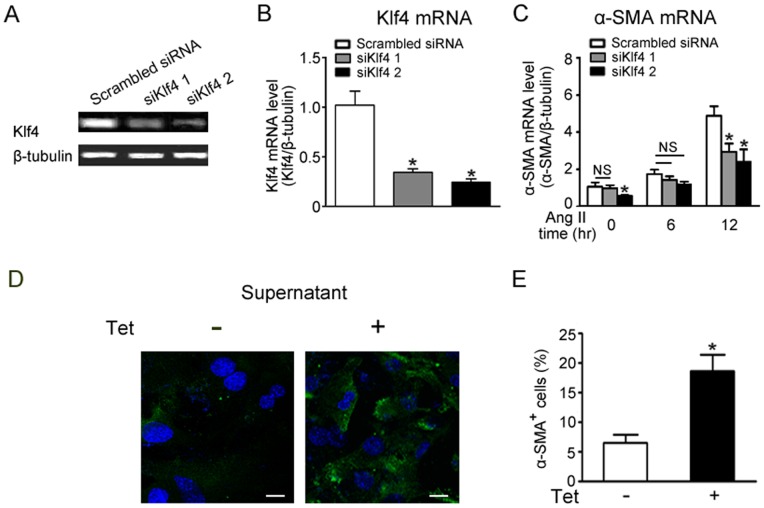
siKlf4 weakens Ang II-induced fibroblast differentiation and collagen synthesis. The effect of Klf4 depends on cytokine secretion. A, semiquantitative PCR of the mRNA levels of Klf4 showed siRNA-mediated knockdown in cardiac fibroblasts. B, Klf4 mRNA levels were assessed by quantitative real-time PCR (qPCR) after siKlf4 transfection (n = 3). C, α-SMA mRNA levels were assessed by quantitative real-time PCR (qPCR) after Ang II treatment for 0, 6 or 12 hrs in siRNA transfected cardiac fibroblasts and normalized to β-tubulin (n = 3). Data are the mean ± SEM. *P<0.05, **P<0.01 vs. scrambled siRNA. D, cultured cardiac fibroblasts were incubated with supernatants collected from cultured cardiac fibroblasts that overexpressed Klf4 or control. Immunofluorescence analysis was performed after staining with α-SMA antibody. E, quantitative analyses of α-SMA positive cells are presented (n = 4). Data are the mean ± SEM. *P<0.05 vs. control supernatant.

When cardiac fibroblasts were transfected with siRNA against Klf4, the level of Klf4 mRNA expression was decreased (Figure 3A&B), whereas Ang II-induced increase in α-SMA mRNA levels was significantly reduced (Figure 3C). Moreover, siRNA against Klf4 also suppressed Ang II-induced collagen genes expression (Figure S3).

To explore if the effect of Klf4 depends on secreting factors, the supernatant from Klf4-overexpressed fibroblasts was collected and added to the primary fibroblasts without any treatment. After 24 hrs, α-SMA positive cells were counted. As shown in Figure 3D, the number of α-SMA positive cells was increased after treatment with the conditional medium from Klf4-overexpressed fibroblasts. These data suggest secreted factors may participate in fibroblast differentiation.

### Klf4 controls Ang II-induced TGF-β1 production

**Figure 4:**
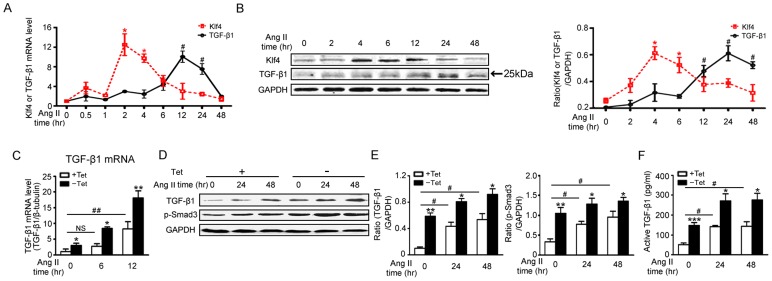
Klf4 controls Ang II-induced TGF-β1 production. A, Klf4 and TGF-β1 mRNA levels were assessed by qPCR in Ang II-treated cardiac fibroblasts and were normalized to β-tubulin (n = 3). B, Klf4 and TGF-β1 protein levels were assessed by Western blot in Ang II-treated cardiac fibroblasts. GAPDH was the loading control (n = 3). Data are the mean ± SEM. *P<0.05, **P<0.01 vs. Ang II treatment for 0 hr. C-F, cardiac fibroblasts were co-infected with Ad-tTA and Ad-Klf4 and treated with Ang II. C, TGF-β1 mRNA levels were assessed by qPCR after Ang II treatment for 0, 6 or 12 hrs and normalized to β-tubulin (n = 4). D, TGF-β1 and phosphorylated Smad3 (p-Smad3) protein levels were assessed by Western blot after Ang II treatment for 0, 24 or 48 hrs. E, level of quantification of TGF-β1 and p-SMAD3 as a ratio of GAPDH in densitometric units was presented. n = 4. F, ELISA was used to analyze active TGF-β1 levels in the supernatant after Ang II treatment for 0, 24 or 48 hrs (n = 5). Data are the mean ± SEM. *P<0.05, **P<0.01 vs. control. ^#^P<0.05, ^##^P<0.01 vs. control and Ang II treatment for 0 hr.

**Figure 5:**
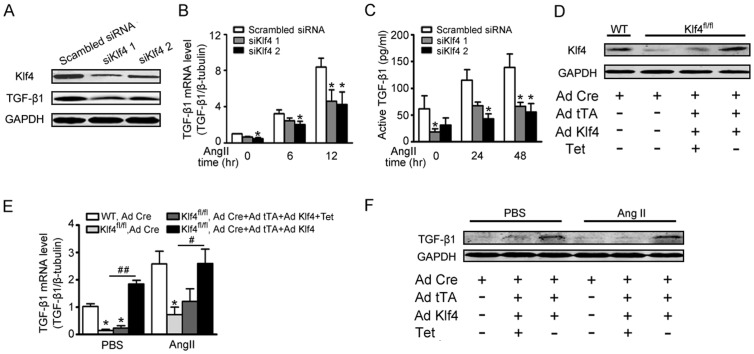
Ang II-induced TGF-β1 production is partly dependent on Klf4. A–C, cardiac fibroblasts were transfected with siKlf4 and treated with Ang II. A, Western blot showed siRNA-mediated knockdown of Klf4 and TGF-β1 protein levels in cardiac fibroblasts. B, TGF-β1 mRNA levels were assessed by qPCR after Ang II treatment for 0, 6 or 12 hrs (n = 3). C, ELISA was used to analyze active TGF-β1 levels in the supernatant of Klf4- or scrambled-siRNA transfected cardiac fibroblasts after Ang II treatment for 0, 24 or 48 hrs (n = 3). *P<0.05 vs. scrambled siRNA. D–F, adult cardiac fibroblasts were isolated from mice carrying LoxP floxed Klf4 alleles. The cells were infected with Ad-Cre for Klf4 deletion, and infected WT cells were served as the negative control. Ad-Cre, Ad-tTA and Ad-Klf4 were co-infected to reintroduce Klf4 expression. D, 2 days after adenovirus infection, the cells were analyzed for Klf4 protein expression by Western blot. E, TGF-β1 mRNA levels were assessed by qPCR after Ang II treatment for 0 or 6 hrs in Klf4-deleted and Klf4-reintroduced fibroblasts and were normalized to β-tubulin (n = 3). Data are the mean ± SEM. *P<0.05 vs. Ad-Cre infected WT control; ^#^P<0.05, ^##^P<0.01 vs. Ad-Cre infected Klf4-floxed fibroblasts. F, TGF-β1 protein levels were assessed by Western blot after Ang II treatment for 24 hrs.

To determine the downstream target genes that are regulated by Klf4 in the fibrotic response, whole genome ChIP-Seq was performed (data not shown). The TGF-β1 promoter region was detected in the sequence of genes that Klf4 could bind. It is known that TGF-β1 secreted by fibroblasts is a pivotal fibrogenic cytokine [26]. Therefore, TGF-β1 could be a Klf4 target gene for fibrosis. To examine the effect of Klf4 on TGF-β1 expression, mRNA and protein expression levels of TGF-β1 were measured. The peak of Klf4 mRNA and protein levels were earlier than TGF-β1 (Figure 4A&B). These data suggest that TGF-β1 could be downstream of Klf4. To establish a direct link between Klf4 and TGF-β1, cardiac fibroblasts were infected with Ad-tTA and Ad-Klf4 and measured for expression of TGF-β1. When Klf4 was overexpressed, TGF-β1 expression was dose-dependent and increased at baseline (Figure S4). The levels of TGF-β1 mRNA and protein were significantly increased in the presence of Ang II (Figure 4C&D). Phosphorylation of Smad3 (p-Smad3), which is known as receptor-associated activating Smad in TGF-β1 signaling, exhibited similar trends as TGF-β1 changes (Figure 4D&E). Moreover, active TGF-β1 was highly enriched in the supernatants that cultured Klf4-overexpessed and Ang II-treated fibroblasts (Figure 4F). Klf4 knockdown significantly reduced the expression of TGF-β1 in fibroblasts (Figure 5A). The Ang II-induced TGF-β1 production was also decreased in fibroblasts knocked down for Klf4 (Figure 5B&C).

To demonstrate whether TGF-β1 production is dependent on Klf4, adult cardiac fibroblasts isolated from LoxP floxed Klf4 mice were infected with Ad-Cre to delete Klf4 (Figure 5D). Similar to siKlf4 interference, expression of TGF-β1 mRNA and protein levels were decreased. When Klf4 was reintroduced by adenovirus, expression of TGF-β1 was recovered (Figure 5 E&F). These similar trends were found for recovered expression of α-SMA, Col1α1, Col1α2 and Col3α1 mRNA (Figure S5).

### Klf4 transactivates the TGF-β1 promoter

**Figure 6:**
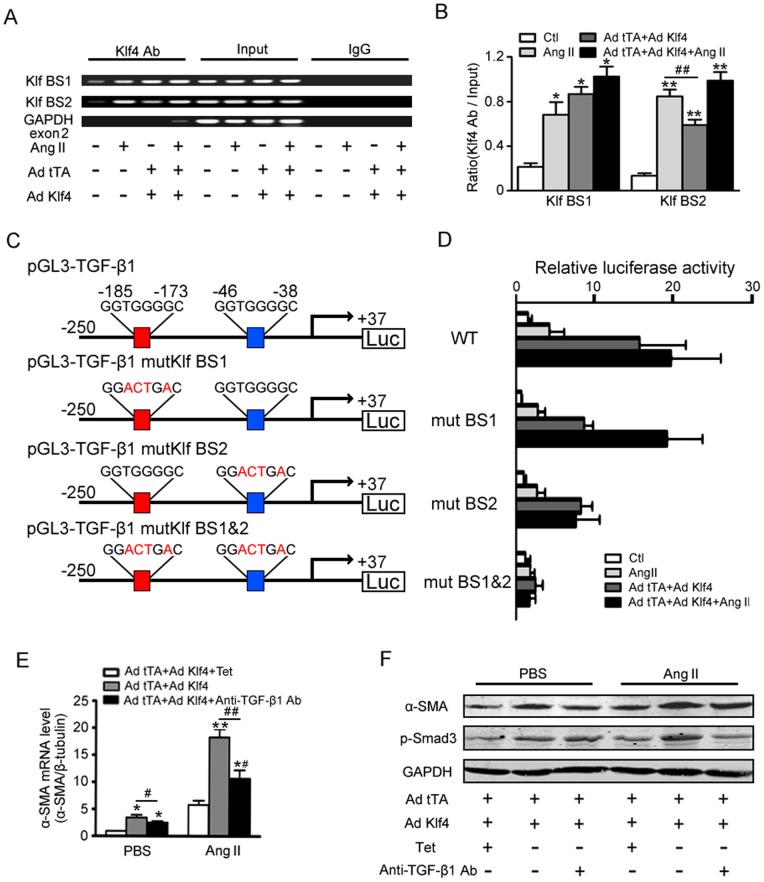
Klf4 transactivates the TGF-β1 promoter. A, ChIP assays of Klf4 binding to the −184∼−180-bp site (Klf BS1) and −45∼−41-bp site (Klf BS1) of the TGF-β1 promoter. A non-target region (GAPDH exon 2) was used as a negative control. B, normalization of each ChIP DNA fraction to the input DNA fraction using densitometric units (n = 3) was shown. Data are the mean ± SEM. *P<0.05, **P<0.01 vs. Ang II untreated and Klf4 non-overexpression. ^##^P<0.01 vs. Ang II treatment. C, pGL3-luciferase reporter driven by the wild-type TGF-β1 promoter or mutant (at −184∼−180-bp and −45∼−41-bp promoter sites) in which the potential Klf4-binding site was constructed. D, promoter-reporter analysis for Klf4-depedent transactivation of the TGF-β1. The pGL3-TGF-β1 WT or mutant vector and CMV-renilla luciferase vector were cotransfected. Luciferase activation driven by the wild-type TGF-β1 promoter or mutant promoter and were normalized to renilla luciferase (n = 4). Data are the mean ± SEM. E-F, effects of neutralizing TGF-β1 on the cardiac fibroblast differentiation activity. An antibody against TGF-β1 (1 μg/ml) was added to the conditional medium. E, the effects of Klf4-dependent α-SMA mRNA expression was assessed by qPCR after Ang II treatment for 0 or 6 hrs (n = 4). Data are the mean ± SEM. *P<0.05, **P<0.01 vs. Klf4 non-overexpression; ^#^P<0.05, ^##^P<0.01 vs. Klf4 overexpression. F, levels of α-SMA and p-Smad3 were assessed by Western blot after Ang II treatment for 24 hrs.

Bioinformatics revealed that the TGF-β1 promoter contains two zinc finger-binding motifs (GTGGG) at −45∼−41 bp and −184∼−180 bp. As shown, Klf4 bound the two sites of the TGF-β1 promoter; a non-target region (GAPDH exon 2) was used as a negative control (Figure 6A). The recruitment of Klf4 to the two sites of the TGF-β1 promoter was enhanced following Ang II treatment at baseline (panel 1 and 2 in Figure 6 A&B), whereas the copies of binding DNA were accumulated when Klf4 was overexpressed (panel 3 and 4 in Figure 6 A&B). At the same time, the −45∼−41-bp site gained higher copies compared with the −184∼−180-bp site by treatment of Ang II, whereas the −184∼−180-bp site exhibited greater binding sensitivity to Klf4 overexpression.

To determine if these two sites are responsible for Klf4-induced TGF-β1 transcriptional regulation, promoter reporter assays were performed using luciferase constructs under control of a 287-bp mouse TGF-β1 promoter with either the normal (WT) or mutated enhancer region (ΔKlf-site 1 or ΔKlf-site 2, as described in Figure 6C). As expected, Ang II strongly enhanced TGF-β1 promoter transcriptional activity (panel 1 and 2 in Figure 6D), and the Ang II-induced promoter activity was enhanced further by Klf4 overexpression (Panel 3 and 4 in Figure 6D). This effect was reduced with mutation of either the −45∼−41- or −184∼−180-bp Klf4-binding site. As mutation of the individual Klf sites conferred a mild reduction in Klf4-mediated transactivation, a more significant loss of activity was observed when both Klf sites were mutated (ΔKlf-1 and ΔKlf-2). Moreover, mutation of ΔKlf-2 (−45∼−41-bp site) influenced promoter activity more obviously than ΔKlf-1 (−184∼−180-bp site) in response to Ang II (panel 2, Figure 6D).

### Klf4 induced TGF-β1 mediates fibrotic responses in cardiac fibroblasts

To determine if TGF-β1 mediates Klf4 effects on fibrosis, a neutralizing antibody was used to inhibit TGF-β1 activity. A neutralizing antibody against TGF-β1 but not control mouse IgG significantly suppressed Ang II-induced α-SMA mRNA and protein expression levels (Figure 6 E&F). Moreover, the phosphorylation of Smad3 was reduced (Figure 6 F).

## Discussion

The present study demonstrated that Klf4 is essential for the differentiation of cardiac fibroblasts to myofibroblasts. Klf4 activates the TGF-β1 promoter activity through classic Klfs binding sites, which leads to increased expression of TGF-β1. These results provide evidence that Klf4 promotes T myofibroblasts differentiation and ECM synthesis at least in part by upregulating TGF-β1 protein.

Hypertensive heart disease is associated with widespread fibrosis [27]–[29]. It is well accepted that the activation of the renin-angiotensin system plays an important pathophysiological role in cardiac remodeling [1], [4]. Once Ang II binds to AT1R, it activates a series of signaling cascades, which, in turn, regulate the physiological effects of Ang II [2]. The array of genes activated by Ang II includes several immediate early transcription factors, such as c-fos, c-jun, c-myc and NF-κB, as well as Egr-1 [8]–[12], [23]. These upregulated transcription factors regulate expression of many genes, such as those for growth factors, cytokines, and adhesion molecules [2], [30]. It is known that Ang II upregulates production and release of TGF-β1 [31]. However, the transactivation mechanism of TGF-β1 is unclear. Hamaguchi et al. showed that Ang II induced an ERK-dependent activation of AP-1, which contributes to TGF-β1 production in VSMCs [13]. ETS-1 has been identified as a mediator of the renal profibrotic effects in the Ang II infusion model through regulation of TGF-β1 [14]. However, there is lack of direct evidence for transactivation.

Members of Klf family transcription factors have been shown to be involved in cardiac remodeling. For example, Klf5 highlights the key role of this family in Ang II and TAC-induced cardiac remodeling [19], [32]_ENREF_19. Klf15 is involved in cardiomyocyte hypertrophy and connective tissue growth factor (CTGF) expression in cardiac fibroblasts [16], [17]. As a member of the zinc finger class of DNA-binding transcription factors, Klf4 (gut Krüppel-like factor; GKLF) was first identified in the epithelial lining of the gut and skin [33]. Klf4 participates in the growth, proliferation and differentiation of many cell types in the cardiovascular system, including VSMCs, endothelial cells, and monocytes/macrophages [34]–[36]. Endothelial Klf4 has been proved to induce an antiadhesive, antithrombotic state and protect against atherothrombosis in mice [37]. Deletion of Klf4 in smooth muscle cells worse the response to vascular injury but accelerate neointimal formation [38]. Despite its wide distribution, critical functions and sensitivity to various cardiovascular diseases, the role of Klf4 in activation of cardiac fibroblasts has not been fully defined. We found that Klf4 was the highest expressed member of Klfs in Ang II-infused mice hearts. The expression of Klf4 mRNA was increased in the early stage of Ang II infusion and maintained at 7 days. Specifically, the expression of Klf4 in cardiac fibroblasts contributes to a majority of the Klf4 increase in Ang II-infused hearts (Figure 1). Importantly, Klf4 participates in Ang II-induced fibrogenic responses, including fibroblast to myofibroblast differentiation and ECM synthesis (Figure 2).

TGF-β1 signaling is a key mediator in cardiac fibrosis by promoting multiple features associated with fibroblast migration, proliferation, and differentiation into myofibroblasts [26]. Our results identified a transcriptional mechanism for Ang II-induced TGF-β1 expression in the cardiac fibroblasts. Our results demonstrated that the expression of TGF-β1 is upregulated by Klf4 through the classic DNA-binding zinc finger motif; the −45∼−41-bp and −184∼−180-bp Klf4 binding site may be weighted differently in TGF-β1 transcription. Affinity binding to the −45–41-bp site is more sensitive than the −184∼−180-bp site by treatment with Ang II (Figure 6).

Experimental results have indicated that Klf4 may be a key effector of SMC phenotypic switching in vascular remolding by SMC gene activation, including the α-SMA gene. It is known that Klf4 has been implicated in VSMCs differentiation induced by TGF-β. For example, the phosphorylated Klf4, formed a Klf4-Smad2 complex in response to TGF-β to bind and activate transforming growth factor-β type I receptor (TβRI) promoter transcription [39]. However Klf4 itself potently represses the expression of multiple SMC genes. Liu et al reported that Klf4 represses SMC genes by both down-regulating myocardin expression and preventing serum response factor/myocardin from associating with SMC gene promoters [34]. Moreover, TGF-β induces activation of α-SMA gene expression in VSMCs at least in part by promoting sumoylation and degradation of the Klf4 protein [40]. Thereby Klf4 regulate α-SMA gene expression indirectly. In this study, we showed that in response to Ang II stimulation, Klf4 regulates TGF-β1 directly and increases α-SMA indirectly, which suggests there are multiple signaling mechanisms that regulate different types of cell differentiation under different physiological and pathological conditions.

Interestingly, neutralizing TGF-β1 couldn't inhibit the effect of Klf4 overexpression (Figure 6E&F). The facts suggest that Klf4 may also regulate other genes than TGF-β1 to promote fibroblast differentiation. Li et al reported that TGF-β increases Klf4 phosphorylation, phosphorylated Klf4 cooperates with Smad to activate the TβRI promoter, leading to a positive feed-back mechanism by which TβRI activation by TGF-β induces the expression and phosphorylation of Klf4, which in turn activates the TβRI promoter. Along with our present study showing that Klf4 binds to TGF-β promoter and increases its expression, the transcriptional factor Klf4 is a key molecule for TGF-β signaling [39]. Further studies are necessary to evaluate these additional TGF-β/ Smads-dependent mechanisms cooperated with Klf4.

Of note, although Klf4 is shown to prompt myofibroblast differentiation in this study, there has been opposite report in TGF-β treated rat lung fibroblast. The results indicated that Klf4-Smad3 interaction inhibited Smad3 binding to the Smad3-binding element in the α-SMA promoter [41]. The opposite verdict indicates the relationship between TGF-β signaling and Klf4 could be more complicated. Although we observe Klf-dependent TGF-β/Smads signaling activation, more feedback and cross-talk mechanisms between TGF-β signaling and Klf4 should be further investigated.

In this study, we unveiled the role of the transcription factor Klf4 as a novel mediator in the activation of profibrotic pathways in the presence of increased activation of the renin-angiotensin system. Given its role as a transcriptional factor to regulate downstream pathways, we have demonstrated that Klf4 transactivates the TGF-β1 promoter and controls its production in Ang II-stimulated cardiac fibroblasts. Through upregulation of TGF-β1, Klf4 mediates Ang II-induced fibroblast differentiation and collagen synthesis, which increases activation of the fibrogenic effect. Taken together, Klf4 may become a target for novel strategies in the prevention and treatment of increased activation of profibrotic pathways in Ang II activation associated end-organ fibrosis.

## Supporting Information

Figure S1 **A–B, Klf expression in cardiomyocytes and VSMCs in response to Ang II infusion.** Klf mRNA levels in cardiomyocytes (A) and VSMCs (B) after 2 hrs of stimulation with PBS control or Ang II (1 μmol/L). Klf8, Klf14, Klf16 and Klf17 were not detectable in cardiomyocytes. Klf1, Klf2, Klf 8, Klf 14 and Klf 17 were not detectable in VSMCs (n = 3). B, Data are the mean ± SEM. *P<0.05, vs. PBS control. C,α-SMA and p-Smad3 protein levels in Ang II-treated cardiac fibroblasts. Protein levels were assessed by Western blot after Ang II treatment for 0, 12, 24 or 48 hrs. GAPDH was a loading control.
(TIF)

Figure S2 **Adenovirus-mediated expression of the VP-Klf4 in cardiac fibroblasts.** A, construction of the tetracycline-regulated adenovirus expressing VP-Klf4. B, cardiac fibroblasts were infected with different titers of Ad-Klf4 and Ad-tTA in the presence or absence of tetracycline (0.1 μg/ml). After 24 hrs, Klf4 protein levels were assessed by Western blot and showed a titer-dependent increase.
(TIF)

Figure S3 **Col1α1, Col1α2 or Col3α1 mRNA levels in siRNA transfected cardiac fibroblasts.** mRNA levels were assessed by quantitative real-time PCR (qPCR) after Ang II treatment for 0, 6 and 12 hrs and normalized to β-Tubulin (n = 3). Data are the mean ± SEM. *P<0.05, **P<0.01 vs. scrambled siRNA.
(TIF)

Figure S4 **TGF-β1 protein levels in cardiac fibroblasts infected with different titers of Ad-Klf4 and Ad-tTA in the presence or absence of tetracycline (0.1 μg/ml).** After infection 24 hrs, TGF-β1 protein levels were assessed by Western blot and showed a titer-dependent increase.
(TIF)

Figure S5 **α-SMA, Col1α1, Col1α2 and Col3α1 mRNA levels in cardiac fibroblasts infected with Ad-Cre for Klf4 deletion or reintroduced Klf4 expression.** mRNA levels were assessed by quantitative real-time PCR (qPCR) after Ang II treatment for 0 and 6 hrs and were normalized to β-tubulin. Panel 1 for Ad-Cre-infected WT cardiac fibroblasts; Panel 2, Ad-Cre-infected Klf4-floxed cardiac fibroblasts; Panel 3, Ad-Cre infected without Klf4 reintroduction; Panel 4, Ad-Cre infected with Klf4 reintroduction (n = 3). Data are the mean ± SEM. *P<0.05, **P<0.01 vs. Ad-Cre infected WT control; ^#^P<0.05, ^##^P<0.01 vs. Ad-Cre infected Klf4-floxed fibroblasts.
(TIF)

Table S1 (PDF)
