# Supplementary figures and images for: Krüppel-Like Factor 4 Transcriptionally Regulates TGF-β1 and Contributes to Cardiac Myofibroblast Differentiation
Source: PLoS One. 2013 Apr 30;8(4):e63424. doi: 10.1371/journal.pone.0063424 (PMC3640021; doi:10.1371/journal.pone.0063424)

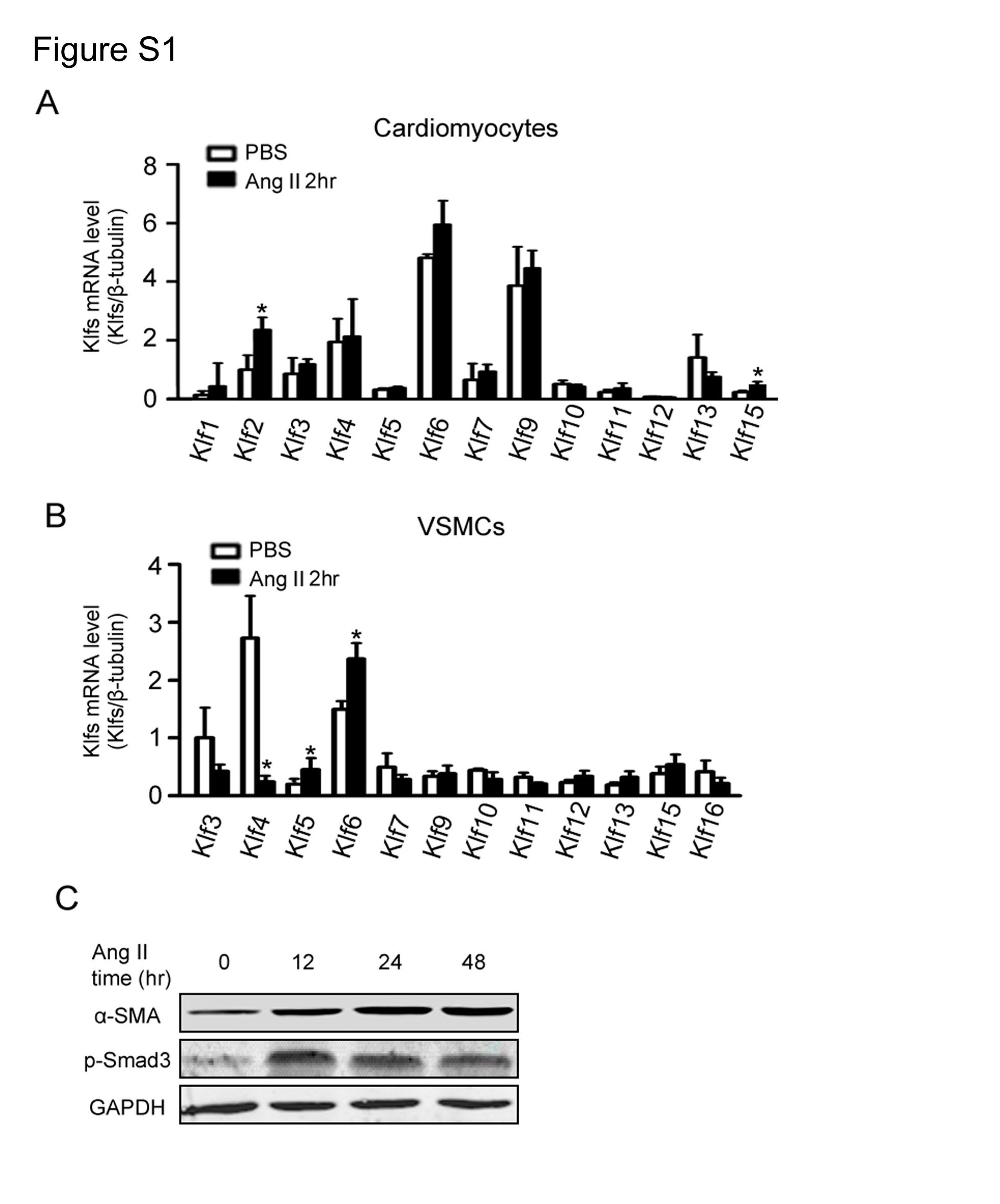

Supplement: Figure S1 — A–B, Klf expression in cardiomyocytes and VSMCs in response to Ang II infusion. Klf mRNA levels in cardiomyocytes (A) and VSMCs (B) after 2 hrs of stimulation with PBS control or Ang II (1 μmol/L). Klf8, Klf14, Klf16 and Klf17 were not detectable in cardiomyocytes. Klf1, Klf2, Klf 8, Klf 14 and Klf 17 were not detectable in VSMCs (n = 3). B, Data are the mean ± SEM. *P<0.05, vs. PBS control. C,α-SMA and p-Smad3 protein levels in Ang II-treated cardiac fibroblasts. Protein levels were assessed by Western blot after Ang II treatment for 0, 12, 24 or 48 hrs. GAPDH was a loading control. (TIF) [file pone.0063424.s001.tif]

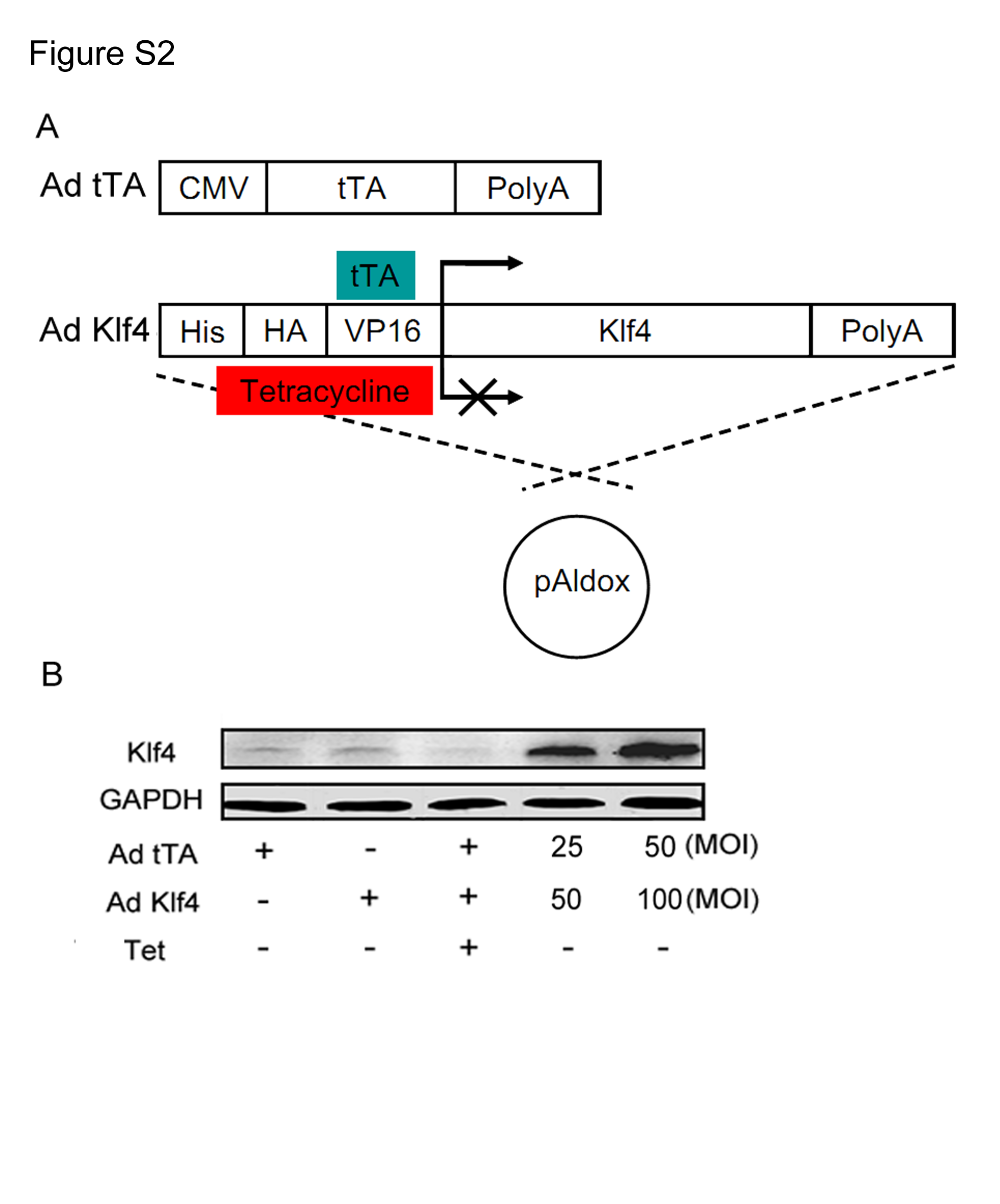

Supplement: Figure S2 — Adenovirus-mediated expression of the VP-Klf4 in cardiac fibroblasts. A, construction of the tetracycline-regulated adenovirus expressing VP-Klf4. B, cardiac fibroblasts were infected with different titers of Ad-Klf4 and Ad-tTA in the presence or absence of tetracycline (0.1 μg/ml). After 24 hrs, Klf4 protein levels were assessed by Western blot and showed a titer-dependent increase. (TIF) [file pone.0063424.s002.tif]

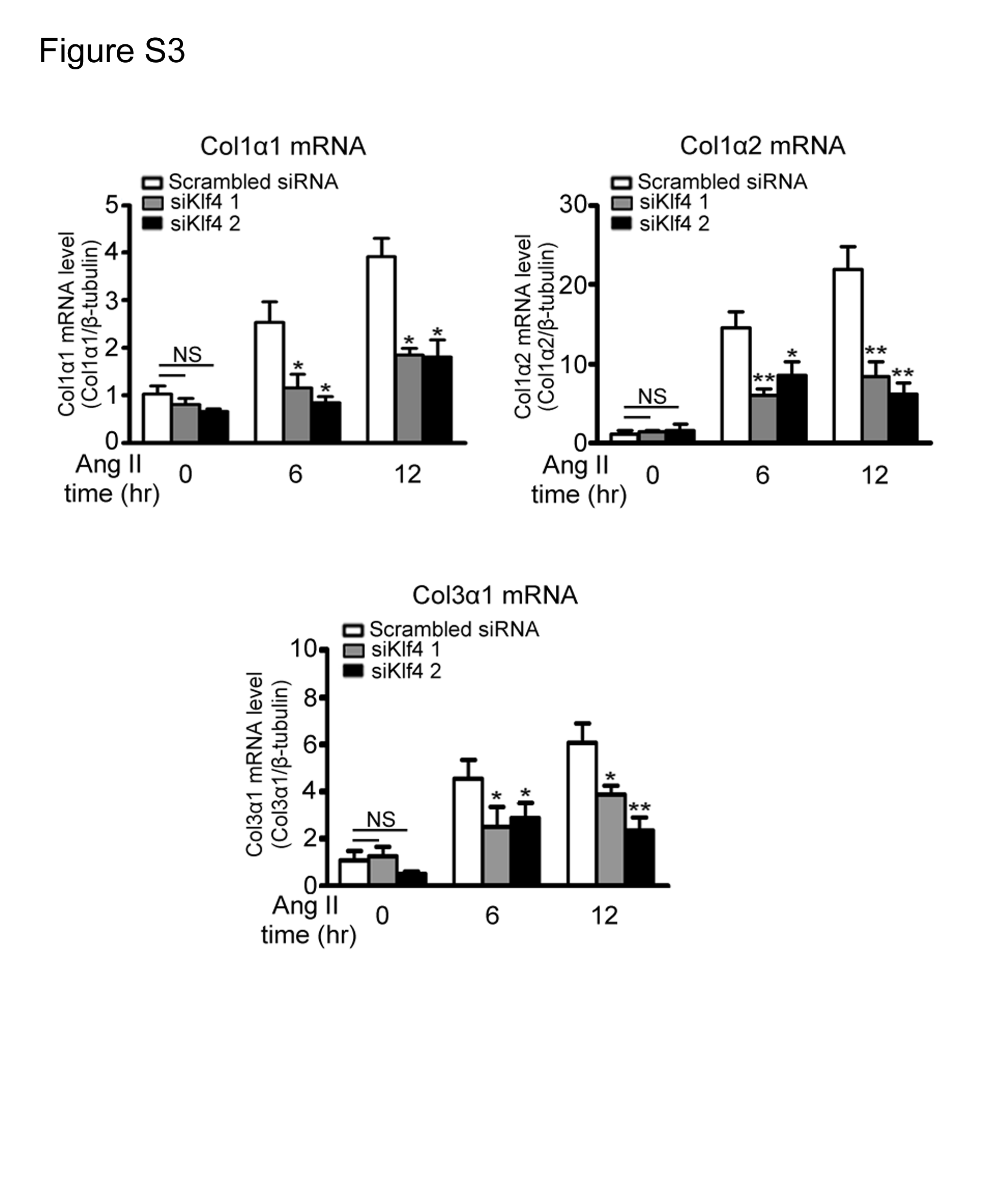

Supplement: Figure S3 — Col1α1, Col1α2 or Col3α1 mRNA levels in siRNA transfected cardiac fibroblasts. mRNA levels were assessed by quantitative real-time PCR (qPCR) after Ang II treatment for 0, 6 and 12 hrs and normalized to β-Tubulin (n = 3). Data are the mean ± SEM. *P<0.05, **P<0.01 vs. scrambled siRNA. (TIF) [file pone.0063424.s003.tif]

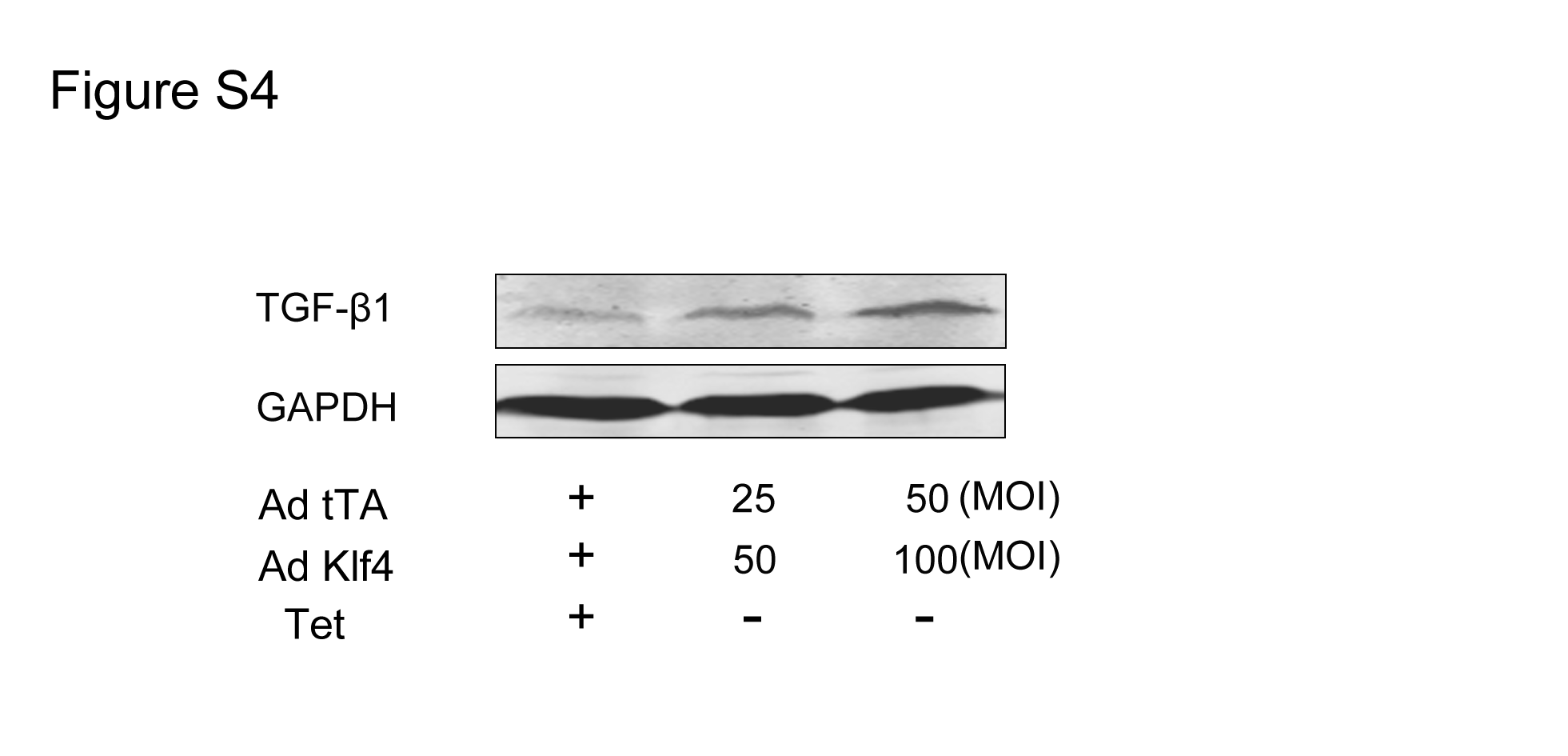

Supplement: Figure S4 — TGF-β1 protein levels in cardiac fibroblasts infected with different titers of Ad-Klf4 and Ad-tTA in the presence or absence of tetracycline (0.1 μg/ml). After infection 24 hrs, TGF-β1 protein levels were assessed by Western blot and showed a titer-dependent increase. (TIF) [file pone.0063424.s004.tif]

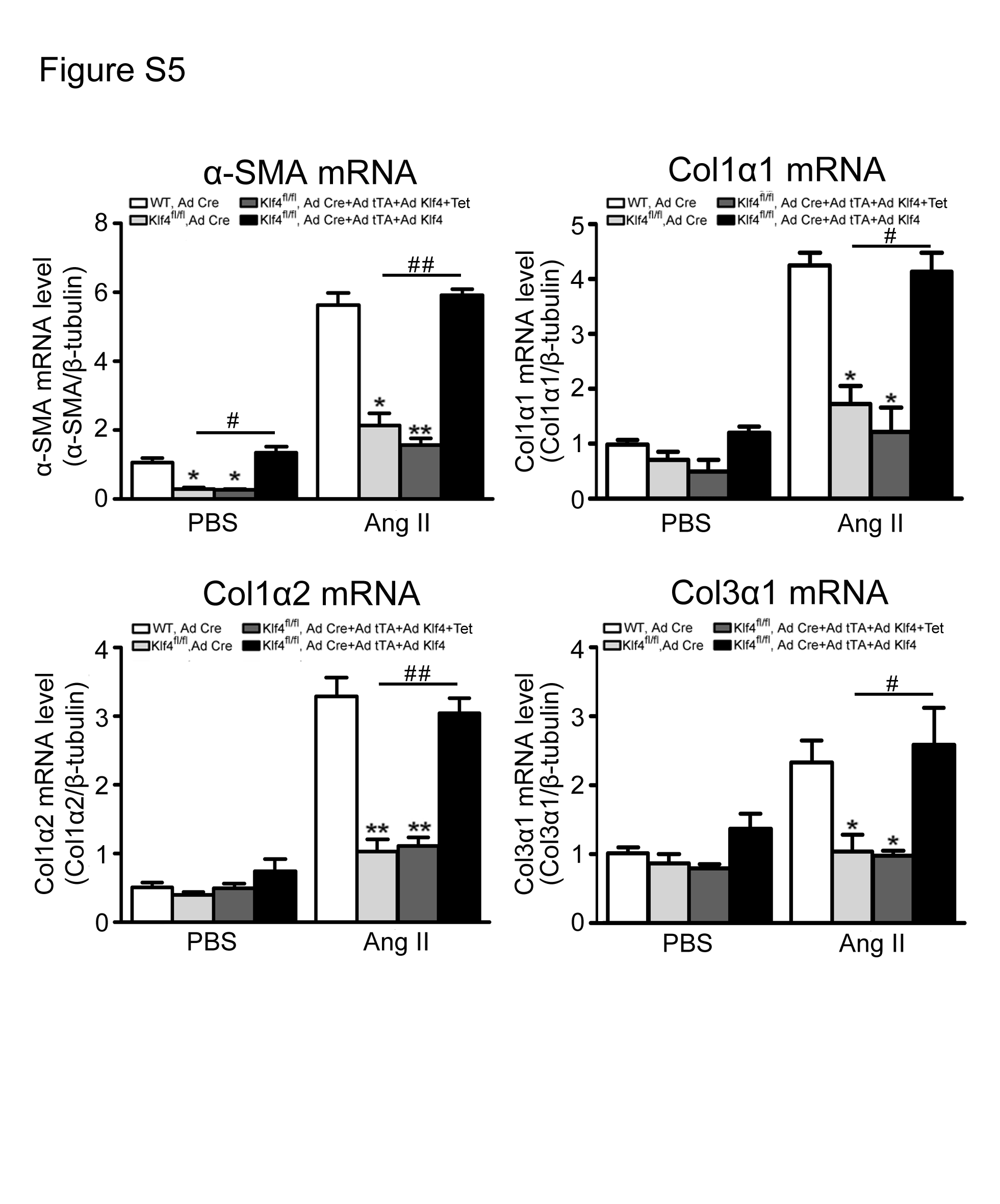

Supplement: Figure S5 — α-SMA, Col1α1, Col1α2 and Col3α1 mRNA levels in cardiac fibroblasts infected with Ad-Cre for Klf4 deletion or reintroduced Klf4 expression. mRNA levels were assessed by quantitative real-time PCR (qPCR) after Ang II treatment for 0 and 6 hrs and were normalized to β-tubulin. Panel 1 for Ad-Cre-infected WT cardiac fibroblasts; Panel 2, Ad-Cre-infected Klf4-floxed cardiac fibroblasts; Panel 3, Ad-Cre infected without Klf4 reintroduction; Panel 4, Ad-Cre infected with Klf4 reintroduction (n = 3). Data are the mean ± SEM. *P<0.05, **P<0.01 vs. Ad-Cre infected WT control; #P<0.05, ##P<0.01 vs. Ad-Cre infected Klf4-floxed fibroblasts. (TIF) [file pone.0063424.s005.tif]
