# Supplementary material for: Krüppel-Like Factor 4 Transcriptionally Regulates TGF-β1 and Contributes to Cardiac Myofibroblast Differentiation
Source: PLoS One. 2013 Apr 30;8(4):e63424. doi: 10.1371/journal.pone.0063424 (PMC3640021; doi:10.1371/journal.pone.0063424)
Supplement: Table S1 — (PDF) [file pone.0063424.s006.pdf]

Table S1

PCR Primer

Gene symbc	Forward primer	Reverse primer	Product size (bp)
Klf1	caagagctcgcacctcaag	gagcgaacctccagtcaca	92
Klf2	ctaaaggcgcatctgcgta	tagtggcgggtaagctcgt	104
Klf3	tcgcacttgaaagcacaca	tcccaggtgcatttgtacg	62
Klf4	cgggaagggagaagacact	gagttcctcacgccaacg	62
Klf5	ccggagacgatctgaaacac	cagatacttctccatttcacatcttg	116
Klf6	tcccacttgaaagcacatca	caaccttcccatgagcatct	68
Klf7	aagtgtaaccactgcgacagg	tcttcatatggagcgcaaga	61
Klf8	gagctccggacacttcagg	catccatgttgtctatgaatttatcc	76
Klf9	ctccgaaaagaggcacaagt	gcgagaactttttaaggcagtc	143
Klf10	agccaaccatgctcaacttc	ggcttttcagaaattagttccatt	76
Klf11	gctcatcttcgcactcacac	tgcgaacttcttgtcacagc	72
Klf12	cactattgttgtaccgctcctg	ttttactctgtctgggagataggc	89
Klf13	caaggcgcacctgagaac	gcgtgcgaacttcttgttg	79
Klf14	aagcgacatcagtgctcctt	tgaggtgcgacgacttgtag	61
Klf15	acaggcgagaagcccttt	catctgagcgggaaaacct	64
Klf16	cacctgcggactcacaca	cagaacgggcgaacttctt	76
Klf17	gagcaggacaataaggaacagg	attcgacactggcaccaac	72
TGF-β 1	tggagcaacatgtggaactc	cagcagccggttaccaag	72
Col1α1	ccgctggtcaagatggtc	ctccagcctttccaggttct	113
Col1α2	gcaggttcacctactctgtcct	cttgccccattcatttgtct	63
Col3α1	tcccctggaatctgtgaatc	tgagtcgaattggggagaat	63
α-SMA	ctctcttccagccatctttcat	tataggtggtttcgtggatgc	60
β-tubulin	cccactccatgtgagtcca	gcaacataaatacagaggtggcta	65
